# New Advertising

**Published:** 1916-12

**Authors:** 


### New Advertising.

The Journal takes pleasure in calling attention to the following new advertising appearing this month, and to commend these firms to the consideration of its readers, believing each of them to be entirely worthy of patronage:

John T. Milliken & Co., Pharmaceutical Chemists, St. Louis, Mo.

F. T. Ramsey & Son, Nurserymen, Austin, Texas.

Marvel Company, Whirling Spray Manufacturers, New York.

Drug Products Company, Manufacturing Chemists, New York.

Farbwerke-Hoechst Company, Medical Preparations, New York.

Iodotone, New York.

T. C. Morgan & Co., Manufacturing Chemists, New York.

The Paul Plessner Co., Manufacturing Chemists, Detaroit, Mich.

It is a pleasure to introduce these firms to the large JOURNAL family and to extend the hope that they may find their association so agreeable and so helpful that they may remain with us permanently.

